# Seismic behavior of precast columns with unbonded prestressed tendons and energy-dissipating bars: physical and numerical investigations

**DOI:** 10.1038/s41598-023-49137-7

**Published:** 2023-12-11

**Authors:** Chao Ma, Xiaobo Zheng, Zhao Zhu, Mi Zhou

**Affiliations:** 1 School of Highway, Chang’an University, Xi’an, 710064 China; 2 Shaanxi Institute of Teacher Development, Shaanxi Normal University, Xi’an, China

**Keywords:** Engineering, Civil engineering

## Abstract

The study of the strength and failure modes of construction components has become increasingly important with the advent of industrialized construction. Recently, because of the need to promote the survivability of both structures and persons, it has encompassed failure modes associated with seismic threats. This study reports the seismic behavior of precast segmented columns designed with both unbonded prestressed tendons (UPTs) and energy-dissipating (ED) bars. A physical specimen was subjected to a quasi-static cyclic test; this permitted the evaluation of the damage mode and behavior of the energy dissipation mechanism, and permitted the establishment and verification of a fiber beam element simulation model. The model was then used to evaluate the effects of the variation of the design parameters on the behavior of precast assembled columns. The test specimen exhibited stable energy dissipation and a pinching effect during cyclic loading. An analysis using the fiber beam element model with varying design parameters revealed that high-strength concrete could mitigate the concrete damage and residual deformation after an earthquake, and that the concrete strength and steel bar strength should be matched when enhancing the seismic resilience of precast columns with high-strength steel bars. It was determined that increasing the prestress mechanism, namely the ratio of the UPTs, could greatly improve the residual deformation, while the ratio of ED bars is the key factor in enhancing the energy dissipation capacity of the precast columns. Suitable ratios of UPTs and ED bars are the keys to ensuring stable energy dissipation and low residual drifts. Moreover, an increased axial load ratio and prestress level are able to restrain the joint opening and improve the bearing capacity of the precast columns; however, an excessive axial load or prestressing force would result in the rapid degeneration of the carrying capacity and larger residual displacements.

## Introduction

To overcome the labor shortage and promote reconstruction after World War II, building industrialization became critical for the reconstruction program in Europe. Related technology achieved high efficiency through standardized design, industrialized prefabrication, and on-site erection. During construction, the structural type, load-transmitting performance, and failure modes of joints are of great significance^1^. To ensure reliable connections in joints, researchers have mainly focused on the construction convenience, flexural or shear properties, and seismic performance of prefabricated architectures^2^. Wet and dry connections are commonly used in precast concrete structures. Wet connections refer to construction with cast-in-place (CIP) concrete after the placement of the linking reinforcements. Because the application of wet connections requires time-consuming on-site operations and curing times, it usually leads to long construction periods^3^. As an alternative to wet connections, unbonded prestressed tendons and anchor bolts have been utilized in dry connections without pouring concrete on-site, thus providing excellent construction convenience and efficiency^4^. However, because the joint behavior is relatively unknown, the characteristics of dry connections have been researched extensively in recent years.

To understand the damage mechanisms and establish design methods using dry joints, experiments and simulations of post-tensioned unbonded prestressed connections have been conducted by many scholars^5,6^. The research results have shown that precast structures with unbonded tendon connections exhibit minor residual deformation and excellent self-centering capacity after earthquakes. Due to the outstanding seismic performance, this method has been suggested as possibly applicable to prefabricated segmented bridge columns.

Because there are differences in the structural function, load characteristics, and reinforcement design of bridges as compared to buildings, the seismic performance of precast bridge columns with unbonded post-tensioned tendon (UPT) connections has been investigated. Perlamo et al.^7^ performed quasi-cyclic tests on UPT connections with a medium drift ratio, and no damage was found to have occurred at a drift ratio of 4.0%. Liu et al.^8^ carried out research on UPT connections with different prestressing degrees. Compared to CIP columns, higher post-yield stiffness, lower residual drift, and reduced (less desirable) energy dissipation were observed. The quasi-static residual displacement and residual crack width were found to be around 66% and 73% of those of reinforced concrete columns, respectively. To define the deformation behavior under seismic ground motions, shaking table tests were employed by Ge and Wang^9^. The results showed that the damage mode was significant flexural cracking that occurred at the joints in the plastic hinge zones, while no cracks were observed in the body of the segmented columns. With a greater shaking magnitude, the opening of the joints became larger, and a swaying phenomenon was observed. Ou et al.^10^ acknowledged that the design must achieve good behaviors during earthquakes, and considered the maximum earthquake conditions; while the results revealed low damage and negligible residual displacement, they also indicated decreased energy dissipation.

To improve the energy dissipation capacity and avoid excessive deformation during earthquakes, steel bars have been used to enhance the damping ratio of the columns. To define the seismic mechanism, some scholars^11,12^ have carried out quasi-static cyclic tests to study the seismic performance of the columns, their makeup, and the methods of application. The research results indicate excellent energy dissipation, self-centering capacity, and low residual displacement with proper reinforcements. Based on existing research, Tong et al.^13^ proposed a connection with UPTs and high-strength energy-dissipating (ED) bars, and the results of experiments revealed good ductile behavior, satisfactory hysteretic energy dissipation, and acceptable self-centering capacity.

Because the design goal of this kind of pier is to take into account self-resetting and energy dissipation, although its seismic performance has been experientially investigated in previous research, the test results have been quite different due to the large differences in the design parameters of the test specimens, and an analysis of the degree of influence of the design parameters is lacking. In this research, based on the results of pseudo-static tests, the skeleton curve, residual offset ratio, and cumulative energy consumption of piers with variations in each design parameter are analyzed. This is of great significance for the identification of the main design parameters affecting the seismic performance and the realization of tough seismic design with both energy consumption and self-resetting capabilities.

However, due to insufficient test results and scarce simulations, a knowledge gap still exists; there is a lack of information about the design parameters and design criteria of precast columns with UPT connections and ED bars. This study partially fills that gap by investigating the seismic performance and design parameters of such columns. To capture the damage mode and hysteresis curve, quasi-static cyclic tests were designed, a physical specimen was constructed, and the tests were performed. The analysis of the measured data, both observed and quantified, permitted the establishment and validation of a fiber beam element model. With the verified model, the design parameters were varied to investigate changes in behavior; the investigated parameters were the concrete strength, steel bar strength, UPT ratio, ratio of ED bars, axial load ratio, and prestress level. The results are meticulously analyzed and discussed, and are of great significance for understanding the seismic responses of, and establishing design methods for, precast columns with UPT connections and ED bars. This information reduces the knowledge gap concerning these structures.

## Experimental results

### Test specimen

**Figure 1:**
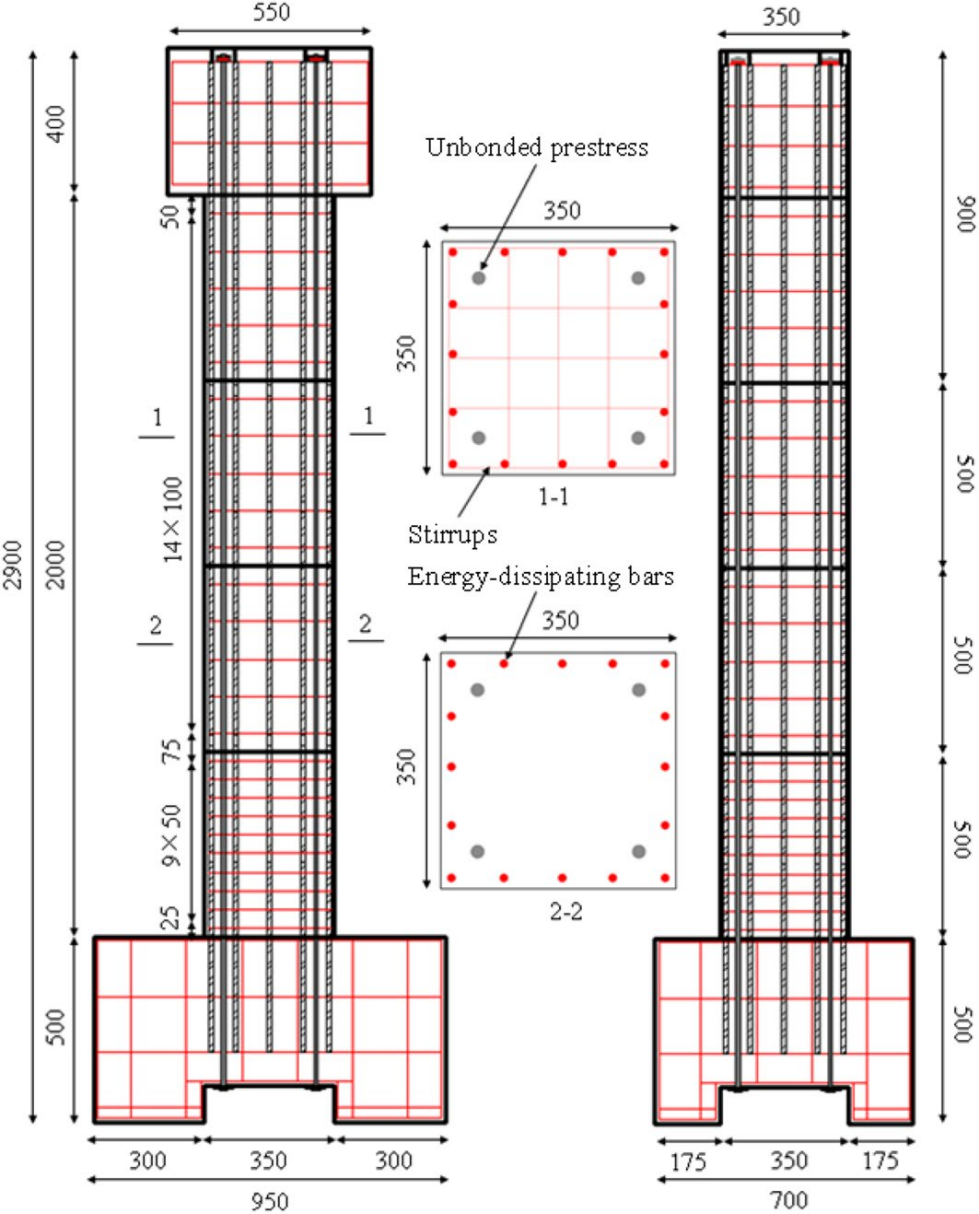
The test specimen (unit: mm).

The physical test specimen was designed according to the Chinese standard, namely *Specifications for Seismic Design of Highway Bridges* (JTG/T 2231-01-2020)^14^. The specimen is depicted in Fig. 1. The total height of the column was 2900 mm, and it was composed of five segments: the footing, the column base (with staples layered at 50 mm), two column segments with staples layered at 100 mm, and the top segment and cap as one unit. The concrete grade was selected as C40, and the total volume was approximately 0.68 m^3^. The steel bars used were HRB400 with a diameter of 12 mm, corresponding to a reinforcement ratio of 1.48%. The diameter of the stirrups was 6 mm, and the volumetric ratio of the bottom segment was 3.13%. The external diameter of the UPT was 15.2 mm, corresponding to a steel ratio of 0.59%, and the ultimate strength was selected as 1860 MPa. In the process of specimen fabrication, because the main reinforcements at the segments were continuous, plastic film was set at the segments during pouring, and the film was removed after construction was completed.

To obtain the real mechanical properties of the material, the compressive properties of the concrete and the tensile properties of the rebar were tested. The concrete specimens used for compressive strength testing were three standard cylinders with a diameter of 150 mm and a height of 300 mm. The compressive strength and elastic modulus of the tested concrete are reported in Table 1, in which $f_{c}{\prime}$ is the compressive strength test values of the specimen, $\overline{f}_{c}$ is the average compressive strength of the specimen, $f_{cu}$ is the conversion of the compressive strength of the cube, where $f_{cu} = \frac{{\overline{f}_{c} }}{0.79}$, *E* is the measured elastic modulus value, and $\overline{E}$ is the average elastic modulus value.

**Table 1:** The concrete material characteristics.

Specimen grouping	Cylinder number	*f*_c_' (MPa)	Average *f*_c_' (MPa)	*f*_cu_ (MPa)	*E* (GPa)	Average *E* (GPa)
I	1	33.9	34.7	43.9	34.3	32.6
	2	38.6			33.5	
	3	31.5			30.1	

The material characteristics of the steel bars are listed in Table 2.

**Table 2:** The reinforcement material characteristics.

Reinforcement type	Diameter (mm)	Average elastic modulus (GPa)	Average yield strength (MPa)	Average ultimate strength (MPa)	Average yield strain	Average ultimate strain
ED bar	12	201.2	429	629.3	0.00226	0.019
stirrup	6	204.3	429.5	547.2	0.00185	0.015
Steel strand	15.2	210.8	1624.5	1993.5	0.00788	0.035

### Test setup and loading protocol

**Figure 2:**
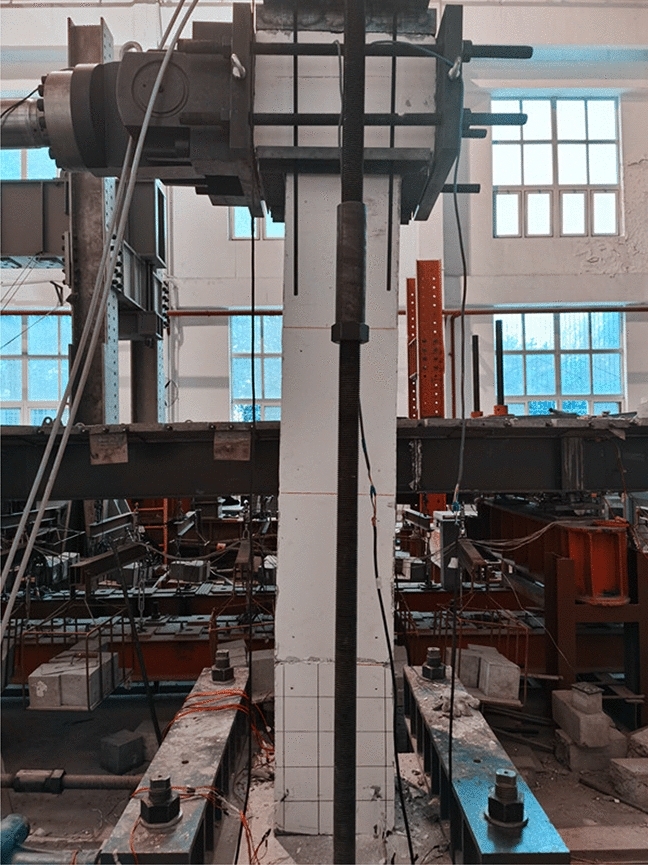
The test setup.

**Figure 3:**
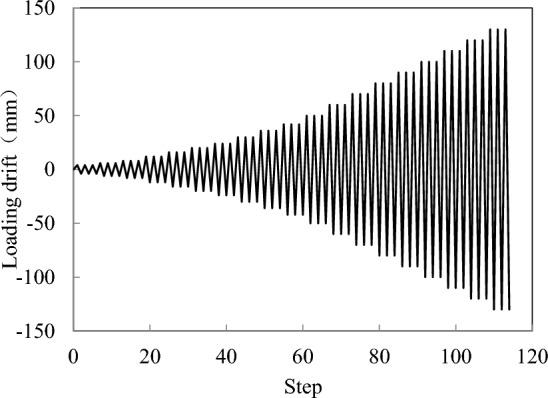
The loading protocol.

To accomplish the experiments, the test setup was arranged as shown in Fig. 2. To ensure a fixed foundation, the footing was anchored by steel beams and bolts. A horizontal actuator was applied in the middle of the cap through the pressing steel plate. The loading height of the column was 2200 mm, corresponding to an aspect ratio of 6.29. By referring to previous experiments^15^, the axial load ratio in this study was determined to be 10%. Therefore, the prestress level was selected as 10%, which implied the tensile force of a single tendon of 56.4 kN. The loading protocol is depicted in Fig. 3. During the test process, displacement-controlled loading was adopted, and each loading stroke was cycled 3 times. When the horizontal force descended to 85% of the maximum force, the cyclic loading was terminated.

### Damage mode

**Figure 4:**
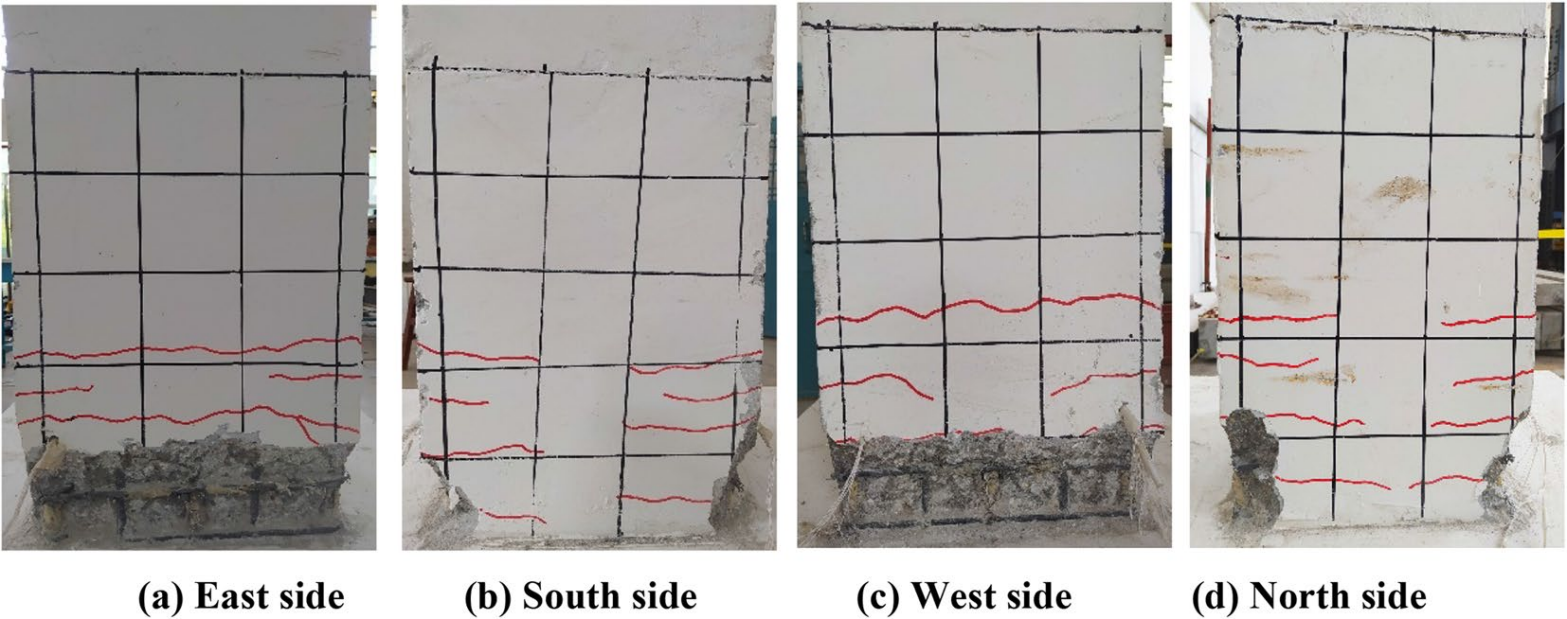
The damage mode of the HBC specimen. (**a**) East side. (**b**) South side. (**c**) West side. (**d**) North side.

**Figure 5:**
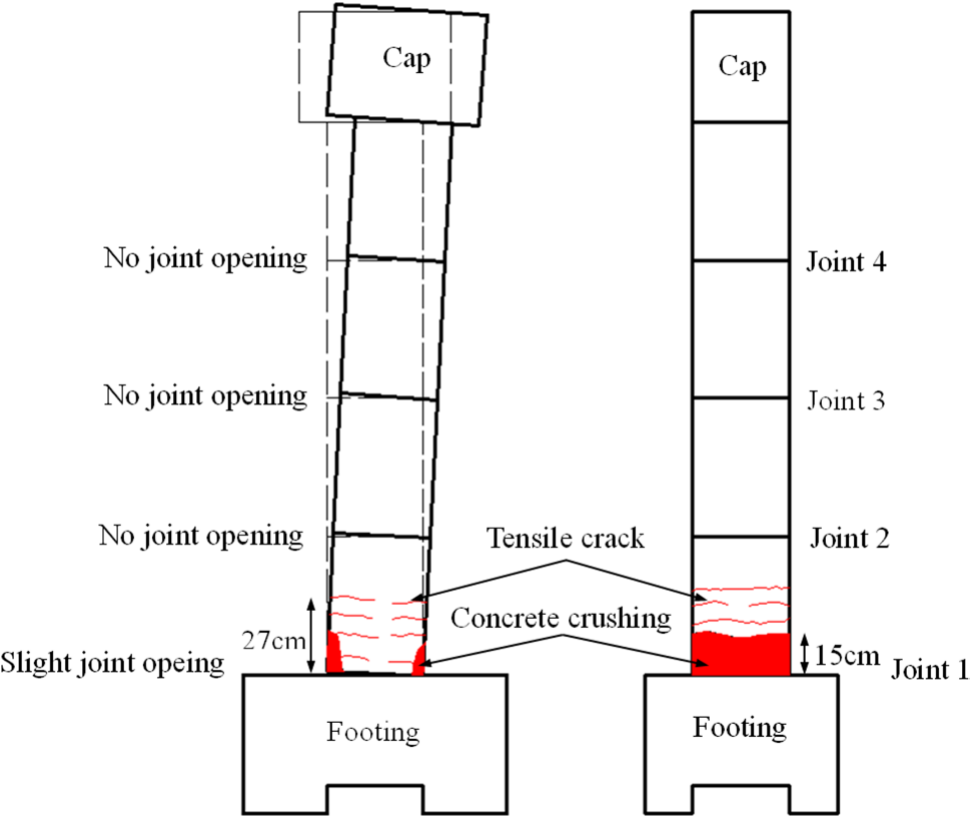
The damage mode.

The final state of the physical specimen is shown in Fig. 4, which depicts the damage mode and damage mechanism. A slight opening of the bottom joint occurred during cyclic loading, and the other joints remained closed. After the cyclic loading ended, concrete crushing and tensile cracks were observed in the bottom segment. The height of the concrete crushing and the fracture distribution were 15 and 27 cm, as shown in Fig. 5.

### Test data

**Figure 6:**
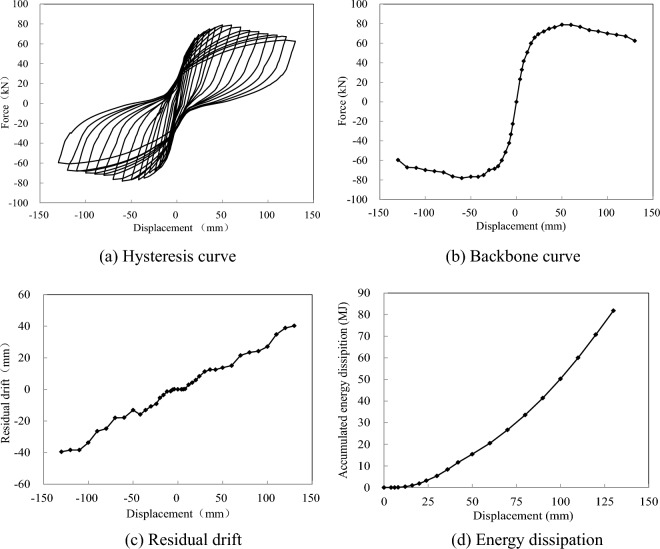
The test data during cyclic loading. (**a**) Hysteresis curve. (**b**) Backbone curve. (**c**) Residual drift. (**d**) Energy dissipation.

The test data comprised a hysteresis curve, a backbone curve, residual drift, and energy dissipation, which were compiled and are depicted in Fig. 6. Compared with a CIP column^16^, the hysteresis curve displayed an obvious pinching effect with stable energy dissipation and lower residual drift. At the displacement of 50 mm, the column responded with a maximum horizontal bearing capacity of 78.94 kN. As the loading continued, the horizontal force descended gradually. When the displacement reached 130 mm, the force descended to 62.50 kN, corresponding to 79.2% of the maximum force, as shown in Fig. 6b. When analyzing the experimental data, a linear relationship was observed between the residual drift and loading displacement. At the end of cyclic loading, the residual drift was almost 40.2 mm, as shown in Fig. 6c. The cumulative energy dissipation was approximately exponentially related to the residual drift, and the total energy dissipation was 81.8 MJ, as shown in Fig. 6d.

## Fiber beam element model and model validation

### Fiber beam element model

**Figure 7:**
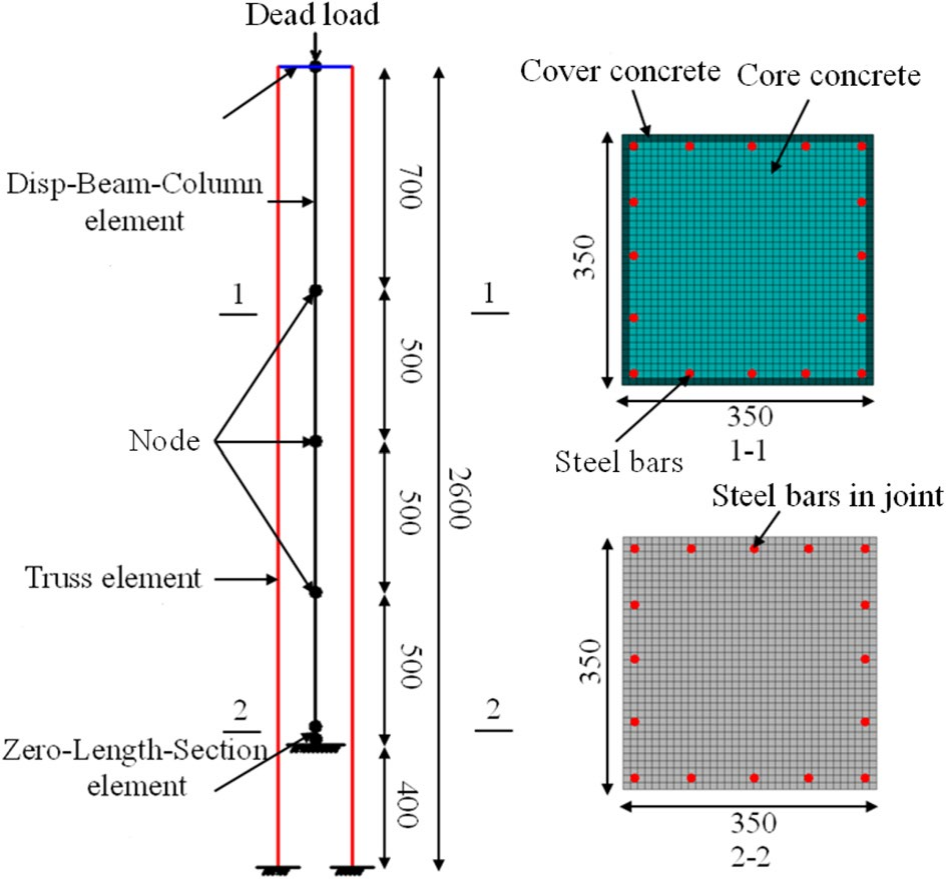
The fiber beam element model.

Based on the test results, a fiber beam element model was established using Opensees software to investigate the design parameters, as shown in Fig. 7. To capture the deformation mode and energy dissipation mechanism during cyclic loading, the disp-beam-column element, the zero-length-section element, and the truss element were selected to simulate the pier shaft, joint, and unbonded prestressed tendon, respectively. In the physical tests, only joint 1 had opened, so only joint 1 was considered in the simulation to improve the computational efficiency.

**Figure 8:**
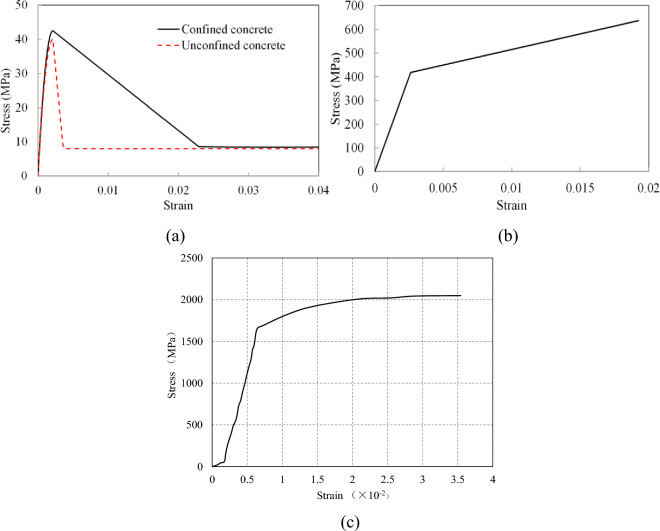
The constitutive law of the materials used in the simulation: (**a**) concrete; (**b**) steel bars; (**c**) unbonded tendons.

To accurately reflect the strain distribution of the concrete, steel bars, and unbonded prestressed tendons, fiber sectioning was utilized, as shown in Fig. 7 (sections 1-1 and 2-2). Considering the confinement effect established by the stirrups, the Concrete 01 model with the Kent–Scott–Park constitutive law^17^ was used to reflect the relative material properties of the cover and core concrete, as shown in Fig. 8a. Furthermore, the Steel 02 model was utilized to describe the bilinear strain/stress curve of the steel bars and unbonded tendons, respectively, as shown in Fig. 8b,c.

### Model validation

**Figure 9:**
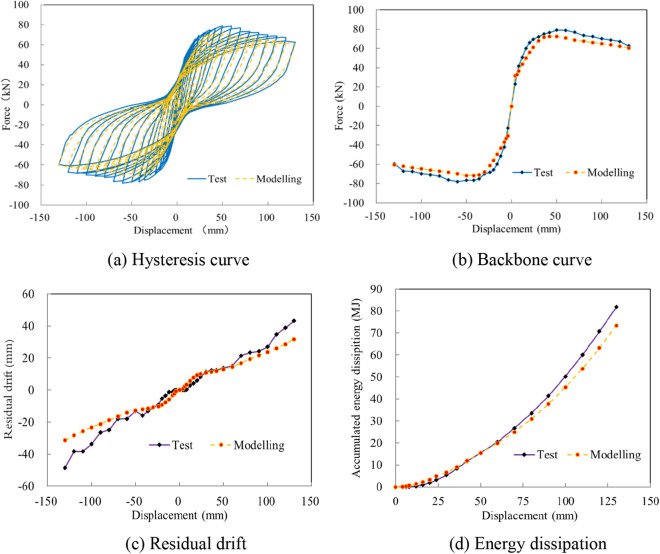
The model validation. (**a**) Hysteresis curve. (**b**) Backbone curve. (**c**) Residual drift. (**d**) Energy dissipation.

As presented in Fig. 9, the measured hysteresis curve, backbone curve, residual drift, and accumulated energy dissipation of the physical experiment were used to validate the numerical model. The model verification results demonstrate that the model could effectively capture the overall energy dissipation capacity and deformation characteristics of the test specimen, as shown in Fig. 9a. The calculated and measured bearing capacities were 72.44 and 78.94 kN, respectively, with a deviation of 8.2%, as shown in Fig. 9b. The hysteresis curve of the analyzed model and the test results exhibited consistent pinching effects, and the residual drifts were respectively 31.7 and 43.2 mm, as pictured in Fig. 9c. Compared with the measured energy dissipation of 81.80 MJ, the calculated energy dissipation was 73.40 MJ, reflecting a reduction of 10.3%, as shown in Fig. 9d. Based on the comparison of the seismic parameters, the simulation was found to reflect the seismic performance of the test specimen.

## Design parameter analysis

Using the verified model, analyses of changes in the characteristics of the precast column with unbonded prestressed tendons and energy dissipation capacity were conducted while varying the several design parameters, and the results were analyzed and discussed. The studied parameters were the concrete strength, reinforcement strength, UPT ratio, ratio of ED bars, axial load ratio, and prestress level.

### Concrete strength

**Figure 10:**
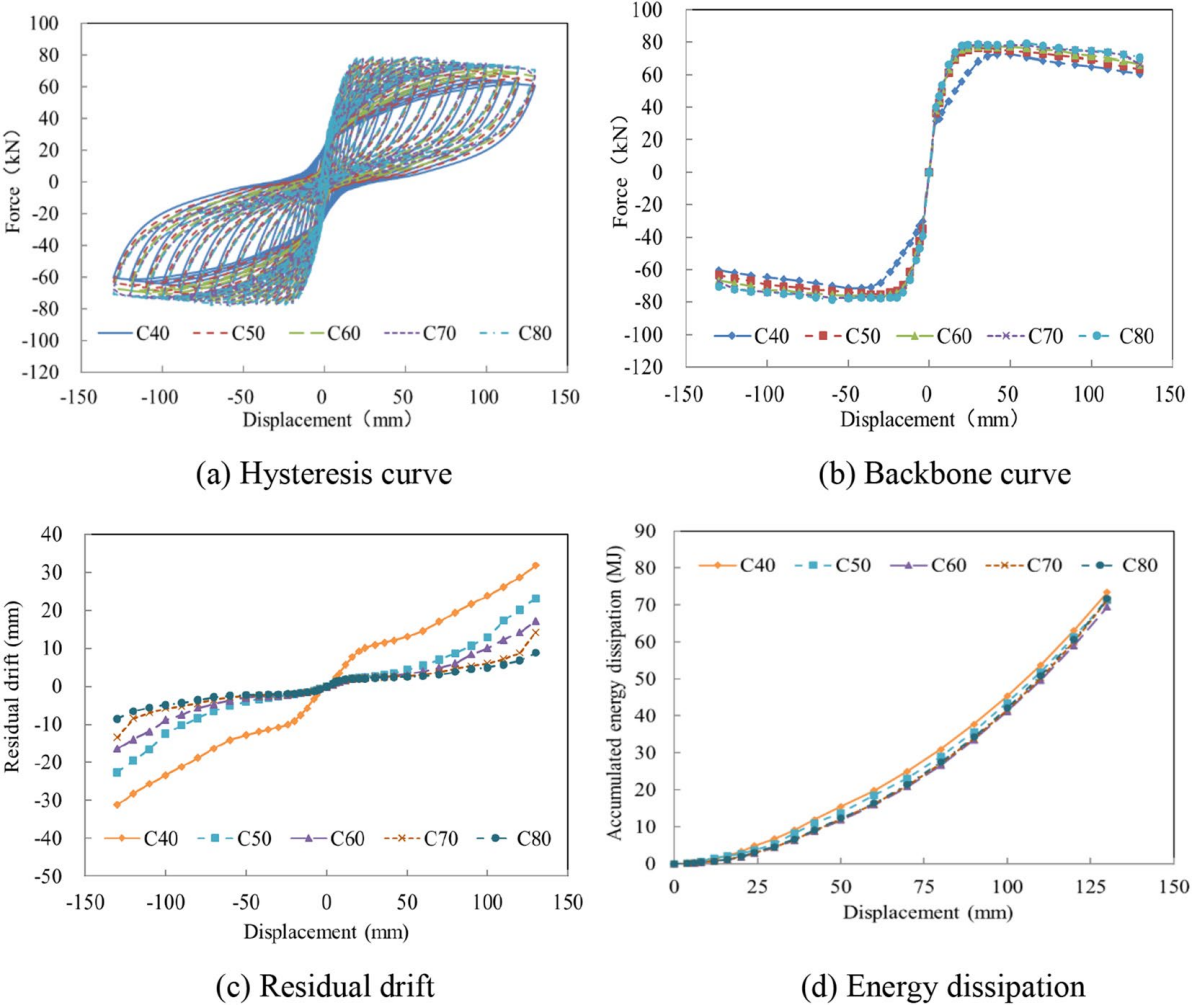
The seismic performance with various concrete strengths. (**a**) Hysteresis curve. (**b**) Backbone curve. (**c**) Residual drift. (**d**) Energy dissipation.

A damage survey showed that concrete crushing is a typical damage mode under earthquake stresses. High-strength concrete has been put forward by scholars^18,19^ to mitigate structural damage and promote rehabilitation after earthquakes. To investigate the seismic performance of the precast column fabricated with various concrete strengths, concrete grades C40, C50, C60, C70, and C80 were analyzed. The research results showed that high-strength concrete enhanced the bearing capacity and initial stiffness, and the obvious pinching effect increased with the grade, as shown in Fig. 10a,b. At the same drift ratio, reduced concrete damage and residual displacement were observed. As the concrete grade increased from C40 to C80, the residual displacement decreased from 31.8 to 8.8 mm, as shown in Fig. 10c. Due to the energy dissipation influenced by the concrete strength, reinforcement ratio, and other factors, the energy dissipation was not improved significantly, as shown in Fig. 10d.

### Steel bar strength

**Figure 11:**
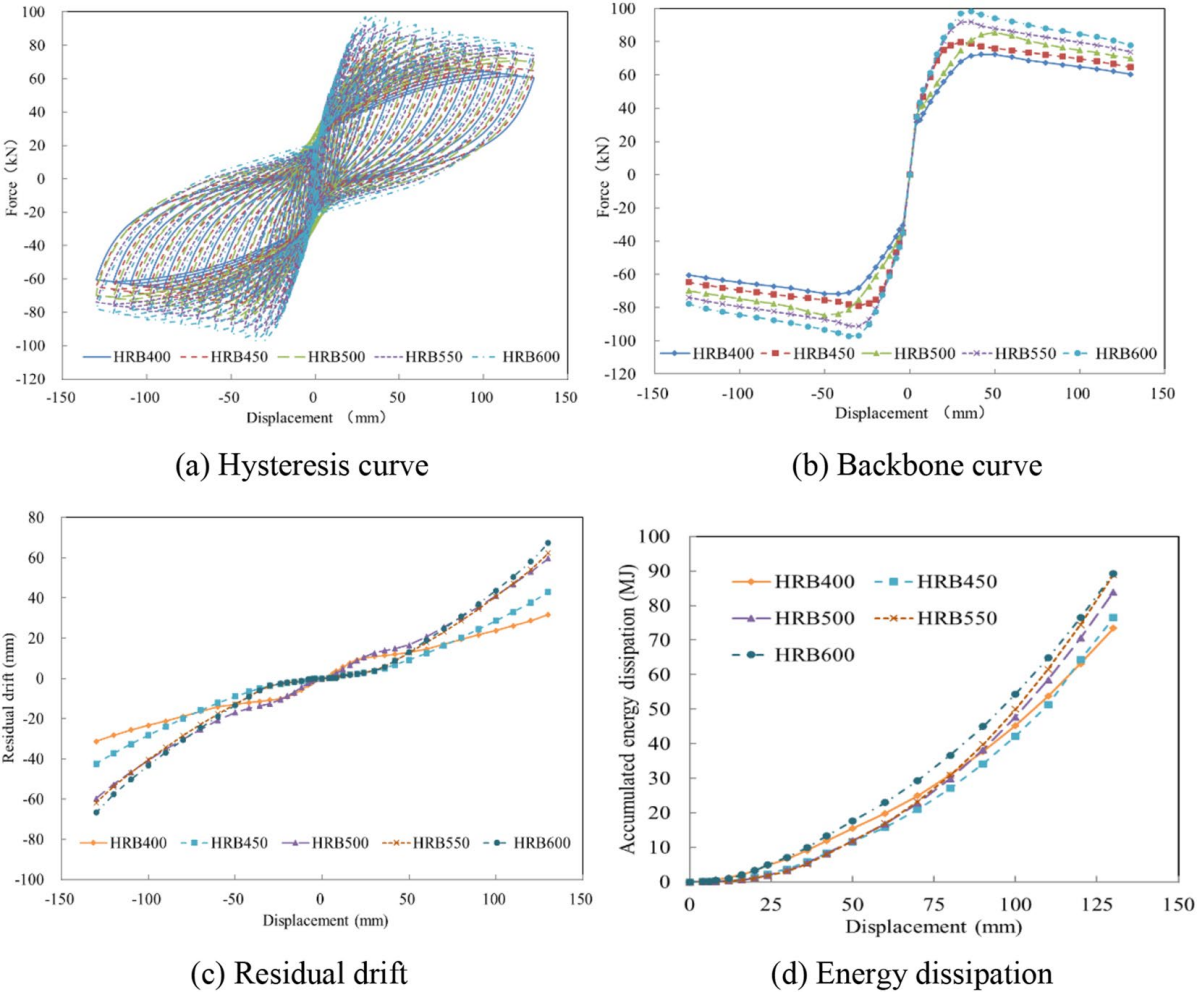
The seismic performance with various steel bar strengths. (**a**) Hysteresis curve. (**b**) Backbone curve. (**c**) Residual drift. (**d**) Energy dissipation.

Regarding concrete crushing, steel bar yielding has been found to be an important failure mode of bridge columns during earthquakes^20^. To study the bearing capacity, residual drift, and energy dissipation of precast columns fabricated with various reinforcement strengths, the steel bar strengths of HRB400, HRB450, HRB500, HRB550, and HRB600 were analyzed and compared, as exhibited in Fig. 11a. The calculation results indicate that the bearing capacity was effectively increased with the application of high-strength steel bars. When the steel bar strength was increased to 600 MPa, the maximum horizontal force was 1.36 times that of the test specimen, as shown in Fig. 11b. However, due to the greater horizontal force, the concrete crushing was aggravated, leading to larger residual displacements, as exhibited in Fig. 11c. With the enhancement of the steel bar strength, the accumulated energy dissipation was slightly improved. When the steel bar strength was 600 MPa, the cumulative energy consumption was 1.21 times that of the column with HRB400, as presented in Fig. 11d.

### UPT ratio

**Figure 12:**
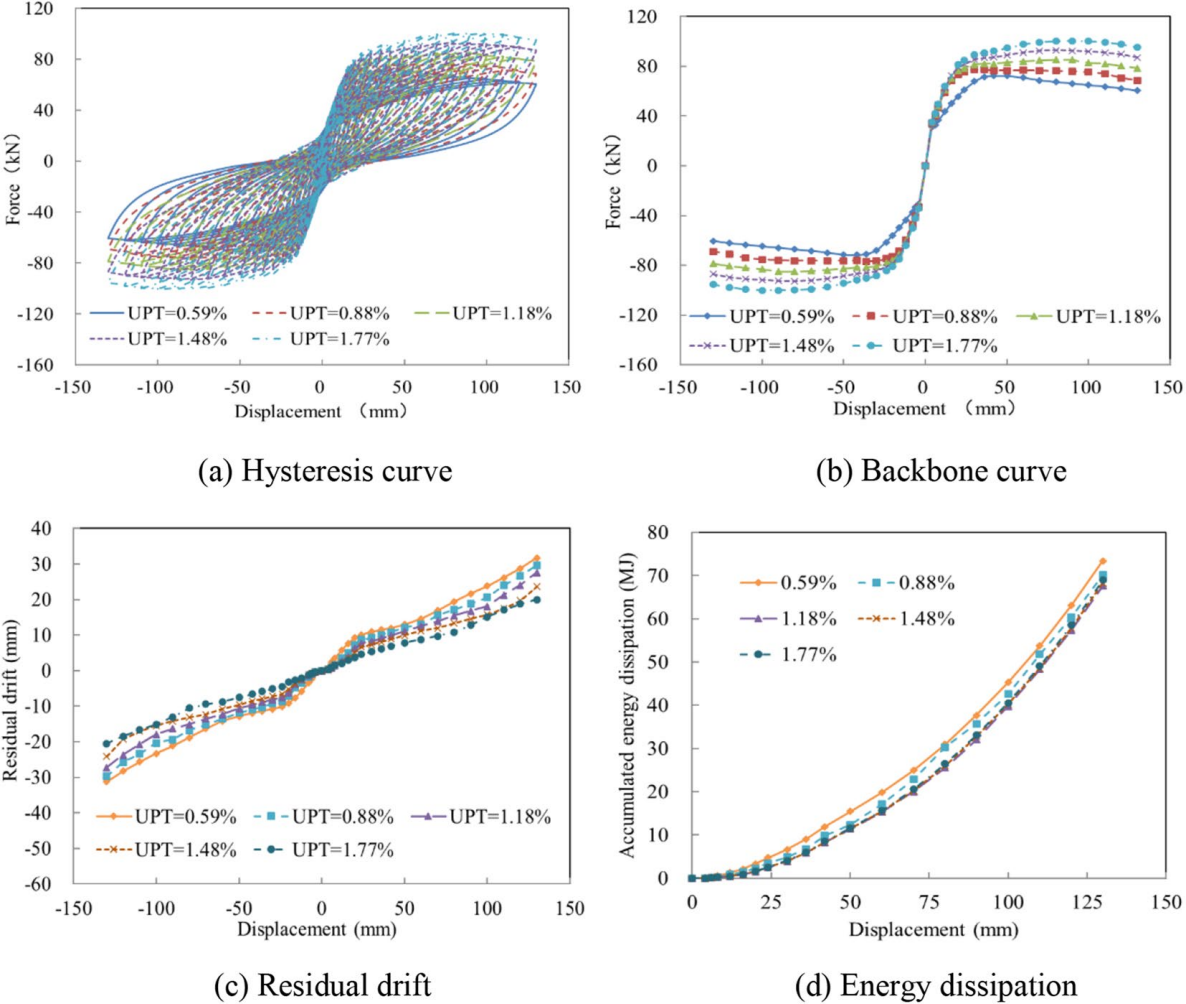
The seismic performance with various UPT ratios. (**a**) Hysteresis curve. (**b**) Backbone curve. (**c**) Residual drift. (**d**) Energy dissipation.

Due to the large free length and effective elasticity of unbonded prestressed elements, they have the capability to supply self-centering capacity during earthquakes. To investigate the seismic performance of columns fabricated with various UPT ratios, the ratios of 0.59%, 0.88%, 1.18%, 1.48%, and 1.77% were selected for investigation and the results were calculated. The bearing capacity was found to be significantly improved with the increase of the UPT ratio, as shown in Fig. 12a,b. Moreover, with the increase of the UPT ratio, the residual drift of the column was gradually reduced. When the UPT ratio reached 1.77%, the residual drift was 20.5 mm, reflecting a 36% reduction as compared to the test specimen, as exhibited in Fig. 12c. As a result of the invariant ratio of the ED bars and the force shared by the unbonded tendons, the cumulative energy dissipation decreased slightly, as displayed in Fig. 12d.

### Ratio of ED bars

**Figure 13:**
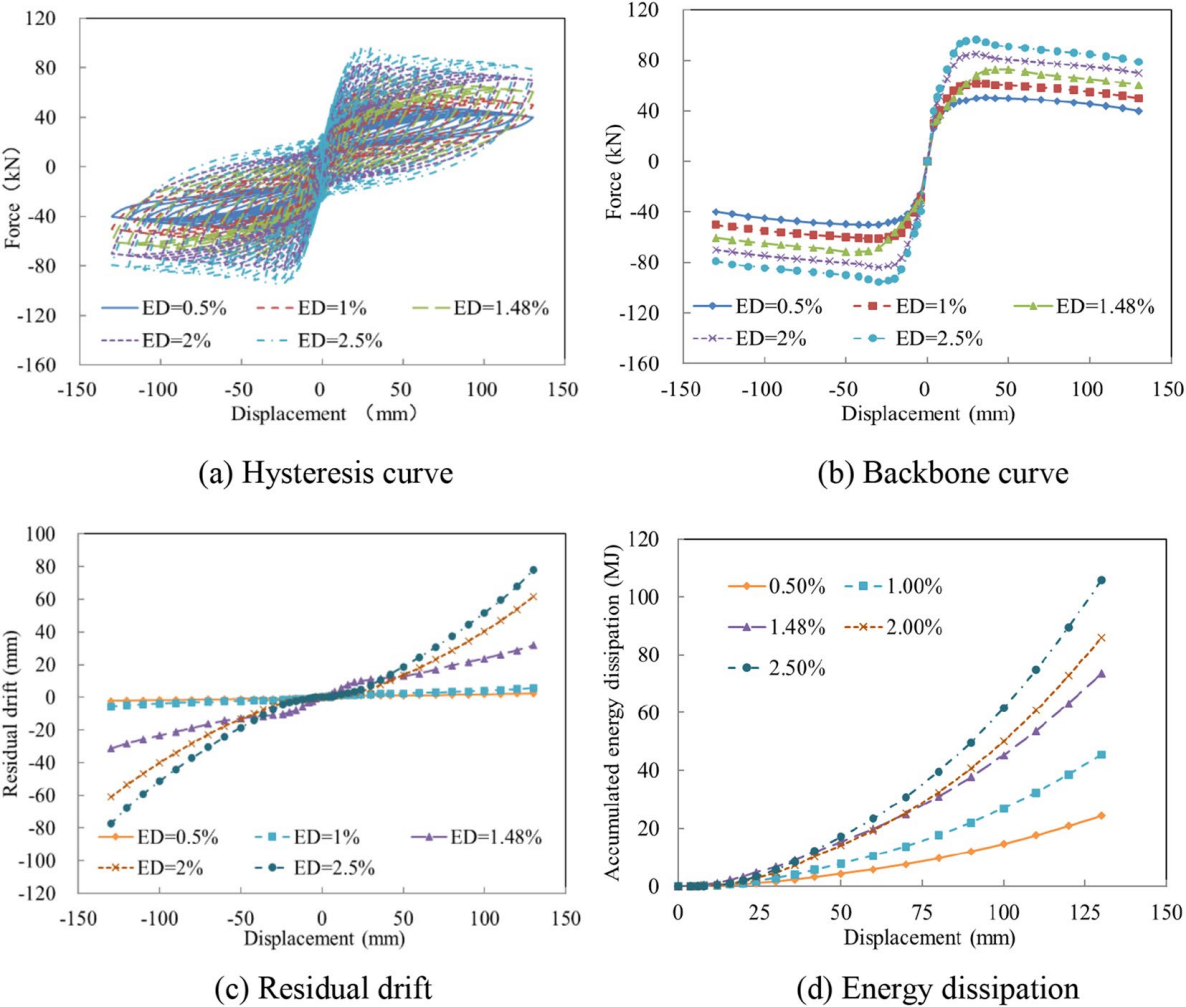
The seismic performance with various ratios of ED bars. (**a**) Hysteresis curve. (**b**) Backbone curve. (**c**) Residual drift. (**d**) Energy dissipation.

The ratio of ED steel bars is one of the most important factors for estimating the energy dissipation capacity of bridge columns. To demonstrate the seismic performance of columns fabricated with various ratios of ED bars, the ratios of 0.5%, 1%, 1.48%, 2%, and 2.5% were selected and analyzed. As the ED bar ratio increased from 0.5 to 2.5%, the hysteresis curves changed significantly, as depicted in Fig. 13a. As the ratio increased from 0.5% to 1%, the precast column exhibited lower energy dissipation and negligible residual displacement. As the ratio increased from 1.48% to 2%, stable energy dissipation and larger residual displacement occurred. Under the same loading displacement protocol, the horizontal force increased significantly as the ratio of ED bars increased, as shown in Fig. 13b. Moreover, with the increase of the ED bar ratio, the residual displacement was magnified, which was obviously due to the large yielding process of the steel bars, as indicated in Fig. 13c. Therefore, a suitable ratio of ED bars was found to be effective for mitigating the residual deformation of the precast column during an earthquake. Figure 13d reveals that the increased ratio of ED bars greatly improved the energy consumption of the column. With the increase of the ED bar ratio from 0.5 to 2.5%, the accumulated energy dissipation rose from 24.35 to 105.79 MJ.

### Axial load ratio

**Figure 14:**
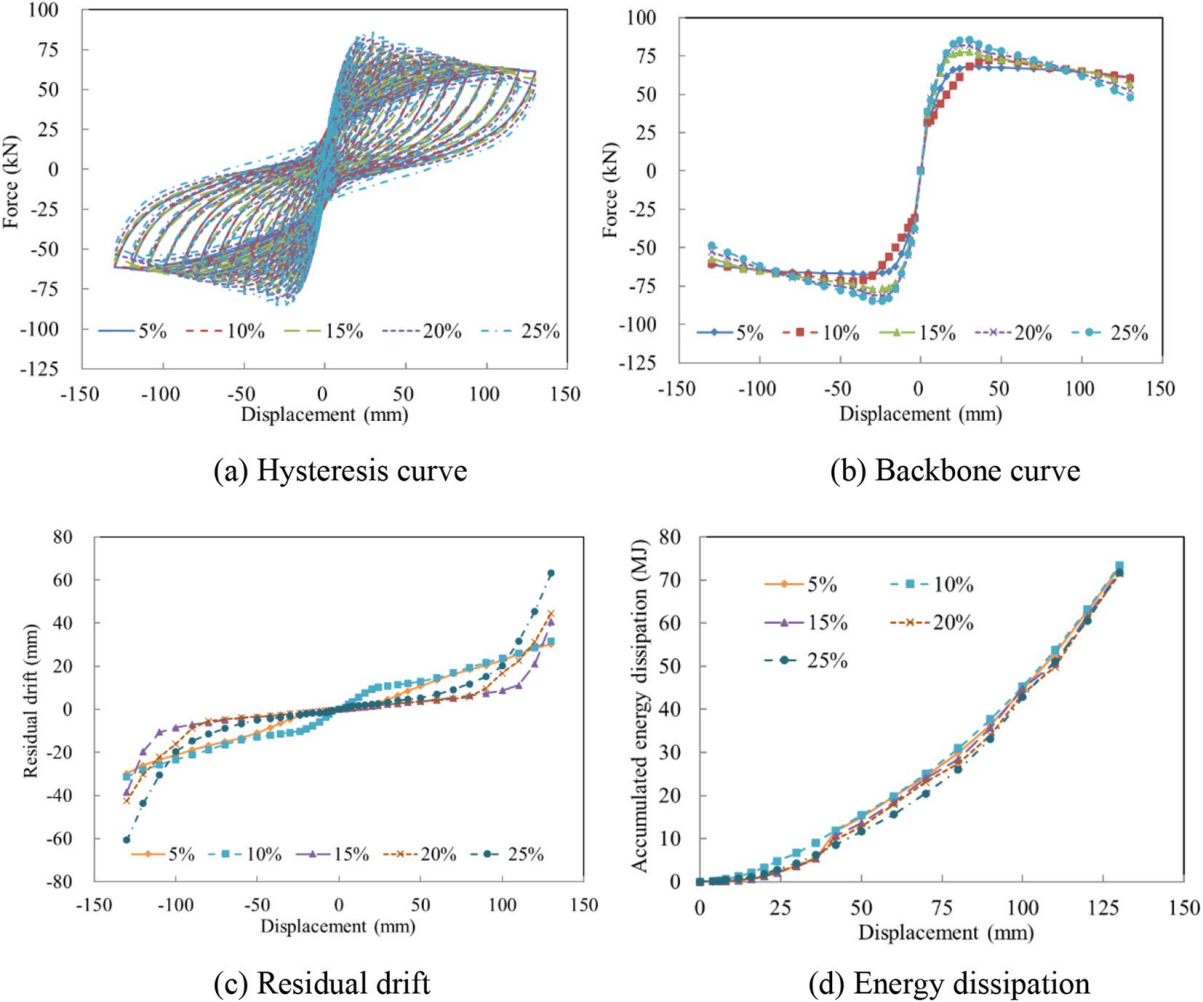
The seismic performance with various axial load ratios. (**a**) Hysteresis curve. (**b**) Backbone curve. (**c**) Residual drift. (**d**) Energy dissipation.

The axial load ratio depends on the material and arrangements of superstructures, and usually ranges from 5 to 25%. The axial load serves to restrain the opening of several joints in a constructed multi-segment column, but high axial loads can also lead to the early failure of concrete in the compression zone. To effectively evaluate the influence of the axial load ratio on the seismic performance of the precast column, columns with various axial load ratios were analyzed. The analysis results reveal that the integrity, initial stiffness, and bearing capacity were enhanced as the axial load ratio was increased, as shown in Fig. 14a. However, the bearing capacity deteriorated rapidly after the horizontal force reached the peak value due to severe concrete crushing in the compression zone, as depicted in Fig. 14b. With the increase of the axial load ratio, more serious concrete damage occurred, resulting in larger residual displacements at the same displacement, as shown in Fig. 14c. Because of the unchanged steel bars, the energy dissipation was enhanced insignificantly, as presented in Fig. 14d.

### Prestress level

**Figure 15:**
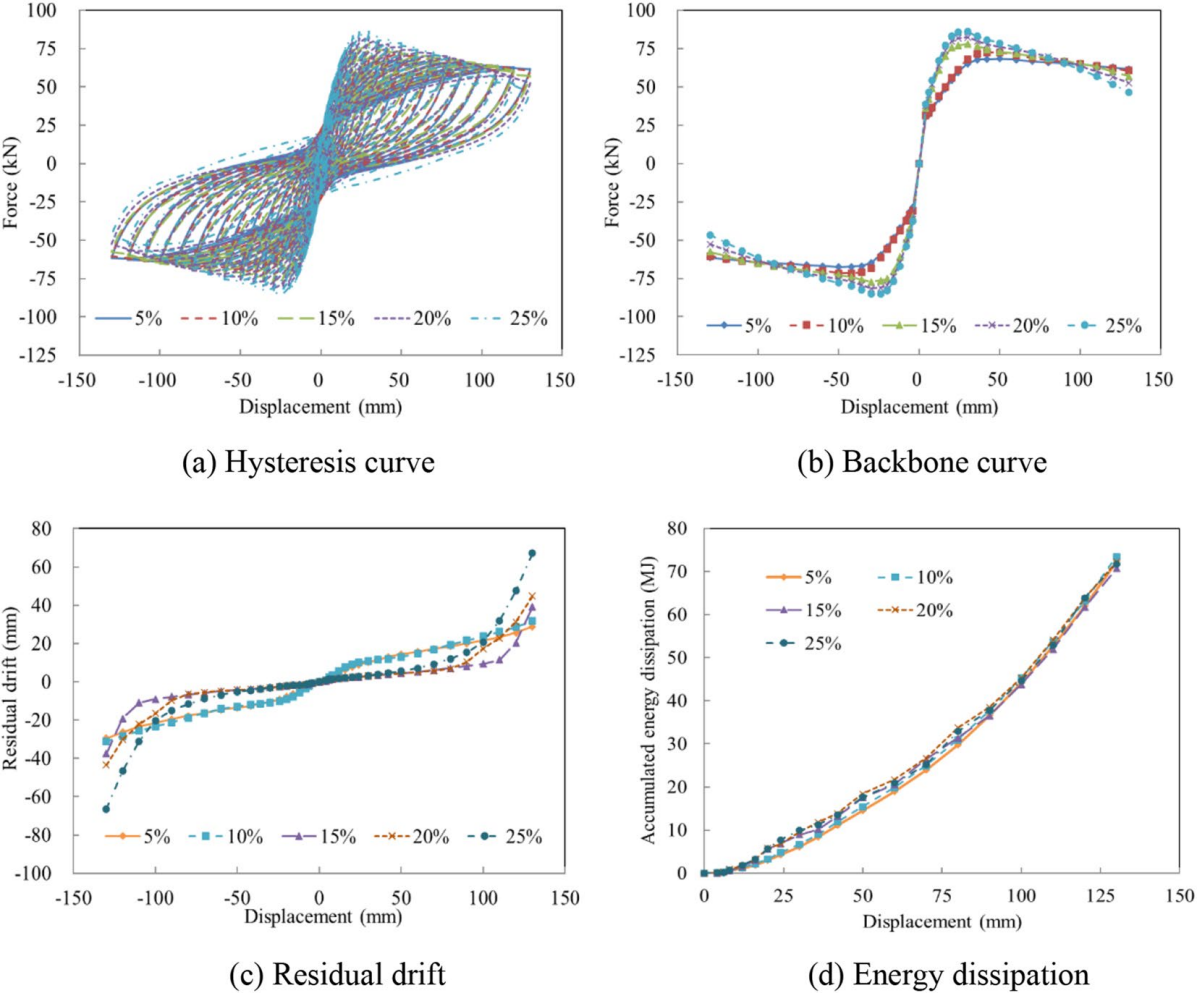
Seismic performance with various axial prestress levels. (**a**) Hysteresis curve. (**b**) Backbone curve. (**c**) Residual drift. (**d**) Energy dissipation.

As with the axial load ratio, the prestressing force of unbonded tendons is a significant design parameter of precast columns. To consider the seismic performance of precast columns under different prestress levels, preload ratios of 5%, 10%, 15%, 20%, and 25% were defined and analyzed. The analysis results reveal similar trends as compared with the axial load ratios. The increase of the prestress level increased the initial stiffness and bearing capacity, leading to larger residual displacements and a minor influence on energy dissipation, as displayed in Fig. 15.

## Conclusion

This study investigated the seismic performance of precast columns with energy-dissipating (ED) bars and unbonded prestressed tendons (UPTs), as influenced by various design parameters. To capture the damage mode and hysteresis curve, force, residual displacement, and energy dissipation of a physical specimen, a quasi-static cyclic test was designed and performed. Using the measured data, a fiber beam element model was established and validated. Using the verified model, the effects of varying the individual design parameters were determined. The design parameters included the concrete strength, steel bar strength, UPT ratio, ratio of ED bars, axial load ratio, and prestress level. The results for each of the design parameters were analyzed and discussed meticulously. The conclusions drawn in this study are as follows.

1. Unlike a simpler precast column with only unbonded tendon connections, the test specimen with ED bars and UPTs exhibited stable energy dissipation and excellent self-centering capacity during cyclic loading. During the experiment, limited rocking was observed due to a slight joint opening that occurred in the bottom joint. The steel bars could not only increase the energy dissipation, but could also inhibit the excessive rotation of the joints. The failure mode of the column exhibited the steel bar yielding and concrete crushing of the bottom joint at a plastic hinge height of 15 cm. After the experiment, the ultimate displacement and residual displacement were 130 and 40.2 mm, respectively, corresponding to drift ratios of 5.9% and 1.8%.
2. The initial stiffness and bearing capacity of the precast column were found to be greatly improved with the dual employment of high-strength concrete and high-strength steel bars. High-strength concrete could mitigate concrete damage and residual deformation after an earthquake. However, when using high-strength steel bars but leaving the concrete strength unchanged, the increased horizontal force will result in severe concrete crushing and excessive residual displacements. Therefore, the concrete strength and steel bar strength should be matched when enhancing the seismic resilience of precast columns with high-strength materials.
3. The increase in the ratios of UPTs and ED bars effectively enhances the resistance of precast columns. The increased ratio of UPTs could greatly improve the residual deformation, but with negligible influence on energy dissipation. The ratio of ED bars was found to be the key factor for enhancing the energy dissipation capacity of the precast columns. However, as the ratio of ED bars increased, the residual deformation deteriorated rapidly. As a result, suitable ratios of UPTs and ED bars were found to be the keys to achieving both stable energy dissipation and low residual drifts.
4. The increased axial load ratio and prestress level are available as design parameters to prevent joint opening and improve the bearing capacity of the precast column. However, excessive axial loads or prestressing force would result in premature crushing failure, the rapid degeneration of the carrying capacity, and larger residual displacements.

## Data Availability

The datasets used in this study were obtained from field tests or finite element model data analysis. The datasets used and/or analyzed are available from the corresponding author upon reasonable request.
